# A comparative analysis of prophylactic antimicrobial use in long-term care facilities in Ireland, 2013 and 2016

**DOI:** 10.2807/1560-7917.ES.2019.24.11.1800102

**Published:** 2019-03-14

**Authors:** Meera Tandan, Rory O’Connor, Karen Burns, Helen Murphy, Sarah Hennessy, Fiona Roche, Sheila Donlon, Martin Cormican, Akke Vellinga

**Affiliations:** 1 Discipline of General Practice, School of Medicine, National University of Ireland Galway (NUIG), Galway, Ireland; 2 Discipline of Pharmacology, School of Medicine, National University of Ireland Galway (NUIG), Galway, Ireland; 3 Health Protection Surveillance Centre (HPSC), Dublin, Ireland; 4 Department of Clinical Microbiology, Royal College of Surgeons in Ireland (RCSI), Dublin, Ireland; 5 Discipline of Bacteriology, School of Medicine, National University of Ireland Galway (NUIG), Galway, Ireland; 6 Department of Medical Microbiology, University Hospital Galway (UHG), Galway, Ireland

**Keywords:** antimicrobial, prescribing, prophylaxis, long-term care facilities, HALT

## Abstract

**Background:**

Long-term care facilities (LTCFs) are important locations of antimicrobial consumption. Of particular concern is inappropriate prescribing of prophylactic antimicrobials.

**Aim:**

We aimed to explore factors related to antimicrobial prophylaxis in LTCFs in Ireland.

**Methods:**

The point prevalence surveys of Healthcare-Associated Infections in Long-Term Care Facilities (HALT) were performed in Ireland in May 2013 and 2016. Data were collected on facility (type and stewardship initiatives) and resident characteristics (age, sex, antimicrobial and indication) for those meeting the surveillance definition for a HAI and/or prescribed an antimicrobial.

**Results:**

In 2013, 9,318 residents (in 190 LTCFs) and in 2016, 10,044 residents (in 224 LTCFs) were included. Of the 10% of residents prescribed antimicrobials, 40% were on prophylaxis, most of which was to prevent urinary tract infection. The main prophylactic agents were: nitrofurantoin (39%) and trimethoprim (41%) for urinary tract (UT); macrolides (47%) for respiratory tract and macrolides and tetracycline (56%) for skin or wounds. More than 50% of the prophylaxis was prescribed in intellectual disability facilities and around 40% in nursing homes. Prophylaxis was recorded more often for females, residents living in LTCFs for more than 1 year and residents with a urinary catheter. No difference in prophylactic prescribing was observed when comparing LTCFs participating and not participating in both years.

**Conclusions:**

Forty per cent of antimicrobial prescriptions in Irish LTCFs were prophylactic. This practice is not consistent with national antimicrobial prescribing guidelines. Addressing inappropriate prophylaxis prescribing in Irish LTCFs should be a key objective of antimicrobial stewardship initiatives.

## Introduction

Antimicrobial resistance (AMR) is a major global concern. The overuse and misuse of antimicrobials in both humans and animals are the leading drivers of AMR [1]. To date, there have been three surveys on Healthcare-Associated Infections in Long-Term Care Facilities (HALT) in the European Union (EU)/European Economic Area (EEA) (2010, 2013 and 2016/17), coordinated by the European Centre for Disease Prevention and Control (ECDC) [2-4]. The second EU/EEA point prevalence survey (PPS) of healthcare-associated infections (HAIs) and antimicrobial use in long-term care facilities (LTCFs), performed in 2013, reported a crude European prevalence of residents on antimicrobials in LTCFs of 4%, ranging from 1% in Hungary to 12% in Greece [2]. In Ireland, the figure was 9.8% and on any given day, residents in Irish LTCFs were twice as likely to receive an antimicrobial compared with the EU/EEA average [5]. A considerable proportion (28%) of antimicrobial use in LTCFs in EU/EEA region was prophylactic [2,3]. In Ireland, the figure was even higher, at 38% [5].

The Irish Health Service Executive (HSE), and the United Kingdom’s National Institute for Health and Care Excellence (NICE) have issued antimicrobial guidelines for various infections both in and outside LTCFs [6,7]. Nevertheless, prophylactic antimicrobial prescribing which is unsupported by evidence or contrary to guidance is widely practised in LTCFs [2,3]. A narrative review of antimicrobial prescribing in nursing homes showed that nearly half of the antimicrobials prescribed were unnecessary [8]. In addition to the societal concerns regarding AMR, inappropriate antimicrobial consumption places patients at risk of serious adverse effects. These include disturbance of normal microbial flora (for example, *Clostridium difficile* infection in the gut, mucosal candidiasis in the skin, etc.) and direct toxic effects of the antimicrobial agent (e.g. pulmonary and hepatic injury related to long-term use of nitrofurantoin) [9,10]. Antimicrobial consumption also places the patient at increased risk of colonisation with antimicrobial resistant organisms which may compromise their subsequent treatment options [11]. Long-term antimicrobial prescribing has also been linked to an increased risk of colorectal adenoma [12]. Therefore, antimicrobial prophylaxis should be limited to specific, well-accepted indications to avoid patient harm and costs, in addition to controlling AMR [13].

The population of Europe is ageing rapidly, with a projected old-age dependency ratio (above 65 years of age) increasing from 28% in 2014 to 50% by 2060 [14]. This ageing population is a major cause of pressure on healthcare systems and costs [15] and is associated with increased demand for nursing homes and LTCF capacity [16]. In 2013, the total LTCF capacity was estimated to be 3.6 million beds in 63,224 facilities for older adults in EU/EEA countries [2]. LTCFs are therefore an important component of healthcare systems in many high-income countries with 2–5% of the older population residing in LTCFs [8]. LTCFs represent a high risk for AMR as they are an environment where immunocompromised individuals live in a communal residence, with a high prevalence of indwelling devices and high antimicrobial consumption [16-18].

Irish HALT survey reports have been published [5,19-21]. This study used data from the 2013 and the 2016 HALT PPS to compare prophylactic use of antimicrobials in Ireland and explores indications and factors related to antimicrobial prophylaxis.

## Methods

### Study design

The HALT PPS was initiated in 2008 by the Healthcare Associated Infections Network (HAI-Net) of the ECDC [2]. The overall aim of HALT was to support the prevention and control of HAIs and reducing antimicrobial use and AMR in the EU/EEA. In Ireland, the HALT PPS was coordinated by the Health Protection Surveillance Centre (HPSC). This study is a secondary analysis of data from the 2013 and 2016 HALT PPS in Irish LTCFs.

### Study setting

The HALT PPS was performed in May 2013 and 2016. The HPSC invited 598 LTCFs in 2013 and 606 LTCFs in 2016 across Ireland to participate in HALT. Participation was voluntary. A standard protocol for conducting each HALT survey was issued by ECDC’s HALT coordinating team and was adapted for local use [22]. Each LTCF participating in HALT in Ireland was required to send one nominated staff member to a regional training day delivered by the HPSC coordinating team. During training, attendees learned how to complete the HALT data collection forms (questionnaires) by practising case studies and were introduced to the survey protocol, methodology and software used.

### Data collection tools

Two separate questionnaires, one institutional and one for residents, were used to obtain information about the LTCF’s characteristics as well as anonymised data on the resident population [5,20]. While both the institutional and resident questionnaire were filled in on the HALT survey date, only the data obtained from resident questionnaires were used for the purpose of this study.

The institutional questionnaire recorded bed occupancy, medical care coordination, infection prevention and control (IPC) resources and activities, the presence of coordinating physicians and antimicrobial stewardship practices. The resident questionnaire was completed only for residents meeting surveillance case definitions for active HAI and/or prescribed systemic antimicrobials. Information on demographics, recent hospitalisations, recent surgery, the presence of vascular or urethral catheters, incontinence, disorientation and impaired mobility were included in the resident questionnaire. Further information was collected on systemic antimicrobial prescriptions, including the name of the antimicrobial, route of administration, therapeutic or prophylactic indication, body site and prescriber occupation.

HAIs were defined using the updated standardised definition; McGeer criteria [23] of infection for surveillance in LTCFs published by the Society for Healthcare Epidemiology of America (SHEA) and the United States Centers for Disease Control and Prevention (CDC) [24].

### Data management and analysis

Within each participating LTCF, data were collected on paper questionnaires and entered into an electronic database using the HALT software. Completed data were subsequently returned to HPSC for analysis. To identify an HAI according to the protocol, data collectors followed the algorithm provided in the resident questionnaire.

Descriptive statistics and multiple logistic regression analysis were carried out in R software v3.4.1 [25]. Data from both PPS combined together and multiple logistic regression were applied for the outcome prophylactic prescribing (prophylactic vs therapeutic) from the residents receiving antimicrobials. Prophylactic prescribing is defined as the prescribing of an antimicrobial with the aim of preventing an infection where residents had no signs or symptoms of infection on the date the antimicrobial was started [26]. Resident’s status of either prophylactic or therapeutic was recorded after reviewing the resident’s medical and nursing notes and/or contacting coordination physicians or general practitioners and/or reviewing the resident’s discharge letter if the antimicrobial was started in hospital. The variables for multiple regression analysis were identified based on previous HALT reports and literature review. The regression analysis used a forward stepwise selection process where each variable was introduced separately and variables with significant change remain in the model. Analysis was performed both including and excluding the missing values in the dataset to see if changes occurred in the outcome, and no differences were observed. The results are presented as odds ratios (OR) and associated 95% confidence intervals (CI). Interactions were tested and omitted from the model if not significant. The p value for the difference in prophylactic prescribing in two PPS for different types of LTCFs was derived from the chi-squared test. A sub-analysis was performed to confirm risk factors for LTCFs that participated in both HALT surveys (2013 and 2016).

## Results

### Prophylactic antimicrobials use by care types

In total, 190 (2013) and 224 LTCFs (2016) participated in the HALT PPS in Ireland. The majority were general nursing homes (GNHs) followed by mixed care, intellectual disability and psychiatric LTCFs. A total of 119 LTCFs participated in both HALT PPS (2013 and 2016), of which 100 LTCFs reported 1,035 residents on an antimicrobial on the day of survey (Table 1). Overall, the total number of participating LTCFs was higher in 2016, but the number of GNHs was lower, from 112 (2013) to 102 (2016). The number of residents was higher in 2016 compared with HALT 2013 (10,044 vs 9,318), an increase of 8% (Table 1).

**Table 1:** Comparison of prophylactic antimicrobial use by care types in HALT, Ireland 2013 and 2016

LTCF types	HALT 2013 (n = 9,318)						HALT 2016 (n = 10,044)						p value^c^
	No of LTCFs	Residents					No of LTCFs	Residents					
		Total	AM^a^		Prophylactic AM^b^			Total	AM^a^		Prophylactic AM^b^		
			n	%	n	%			n	%	n	%	
**Participated in both surveys (119 LTCFs)**													
GNH	58	2,881	314	10.9	120	38.2	54	2,725	235	8.6	104	44.3	0.18
Mixed facility	26	1,447	79	5.5	32	40.5	24	1,397	149	10.7	48	32.2	0.27
IDF	19	833	72	8.6	38	52.8	19	892	61	6.8	30	49.2	0.81
Psychiatric	7	200	10	5.0	4	40.0	7	173	10	5.8	5	50	0.65
Palliative	4	90	30	33.3	10	33.3	4	93	30	32.3	5	16.7	0.23
Rehabilitation	3	181	14	7.7	2	14.3	3	187	12	6.4	3	25.0	0.49
Physical disability	1	28	0	NA	NA	NA	1	13	0	NA	NA	NA	NA
Others	1	29	0	NA	NA	NA	7	177	19	10.7	11	57.9	**NA**
**Total (both surveys)**	**119**	**5,689**	**519**	**9.1**	**206**	**39.7**	**119**	**5,657**	**516**	**9.1**	**206**	**39.9**	**0.99**
**Participated in one or both surveys (190 in 2013 and 224 in 2016)**													
GNH	112	6,019	567	9.4	217	38.3	102	5,163	493	9.5	213	43.2	0.10
Mixed facility	32	1,571	165	10.5	56	33.9	46	2,499	250	10.0	106	42.4	0.08
IDF	24	1,060	106	10.0	54	50.9	31	1,251	102	8.2	55	53.9	0.66
Psychiatric	11	345	23	6.7	6	26.1	23	505	39	7.7	12	30.8	0.69
Palliative	4	89	31	34.8	10	32.3	7	134	44	32.8	9	20.5	0.24
Rehabilitation	3	139	14	10.1	2	14.3	5	245	22	8.9	5	22.7	0.53
Physical disability	2	46	0	NA	NA	NA	1	13	0	NA	NA	NA	NA
Others	2	49	7	14.3	5	71.4	9	234	31	13.2	17	54.8	0.42
**Total (one or both surveys)**	**190**	**9,318**	**913**	**9.8**	**350**	**38.3**	**224**	**10,044**	**981**	**9.8**	**417**	**42.5**	**0.06**

AM: antimicrobials; GNH: general nursing homes; HALT: healthcare-associated infections in long-term care facilities; IDF: intellectual disability facility; LTCF: long-term care facility; NA: not applicable.

^a^ Denominator for percentages is total residents in the facility.

^b^ Denominator for percentages is AM.

^c ^p value of difference in prophylaxis between 2013 and 2016.

The prevalence of antimicrobial prescribing was similar in 2013 and 2016. However, in 2016 the proportion of prophylactic antimicrobials prescribed seemed to be higher (42%) compared with 2013 (38%). However, when only comparing LTCFs that participated in both surveys, the prophylactic prescribing was the same for both surveys (i.e 40%). Overall antimicrobial prescribing was higher among GNH residents compared with any other LTCF types in both HALT surveys, while the proportion of residents receiving prophylaxis was higher among residents in intellectual disability facilities (IDFs) for all LTCFs, as well as for the 119 LTCFs that participated in both surveys (Table 1). No significant differences were observed from 2013 to 2016 for prophylactic prescribing by facility type.

### Indication of prophylactic antimicrobials by body sites

In both surveys, of all prophylaxis prescribed (n = 350 in 2013 and n = 417 in 2016), the urinary tract accounted for the majority (75% and 70% respectively), followed by respiratory tract (12% and 18%), skin or wounds (9% and 8%) and other sites (4% and 4%) (Not shown in table). Similarly, urinary tract accounted for the majority (72% in 2013 and 71% in 2016) of all prophylaxis prescribed (n = 206 in 2013 and n = 206 in 2016) for the 119 LTCFs that participated in both surveys. Prophylaxis prescribing for respiratory tract was higher in 2016 (14%) compared with 2013 (9%) but less for skin or wounds in 2016 (9%) compared with 2013 (12%).

**Figure 1:**
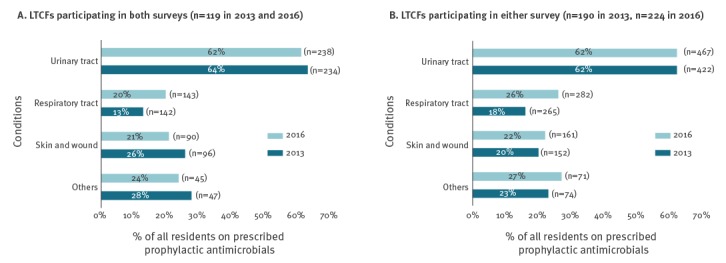
Proportion of residents prescribed prophylactic antimicrobials, by body site targeted, healthcare-associated infections in long-term care facilities point prevalence surveys, Ireland, 2013 and 2016, (A) in long-term care facilities which participated in both surveys (n=119) and (B) in long-term care facilities which participated in either survey (n = 190 in 2013 and n = 224 in 2016)

Analysis of the 119 LTCFs that participated in both surveys showed higher prophylactic prescribing in 2016 (20%) than in 2013 (13%) for respiratory tract (Figure 1A). Similarly, the proportion of prophylaxis was higher in 2016 (26%) than in 2013 (16%) for the respiratory tract of all antimicrobials prescribed, both prophylactic and therapeutic (n = 265 in 2013 and n = 282 in 2016). Of all antimicrobials prescribed specifically for the urinary tract, as well as for skin or wounds, the proportion prescribed as prophylaxis remained the same for both years (Figure 1B). Prophylaxis for other indications was higher in 2016 (27%) compared with 2013 (23%) (Figure 1B).

n refers to the total number of residents on antimicrobials (both prophylactic and therapeutic) for a specific condition during the survey.

### Prophylactic prescribing of antimicrobial agents by body site

**Figure 2:**
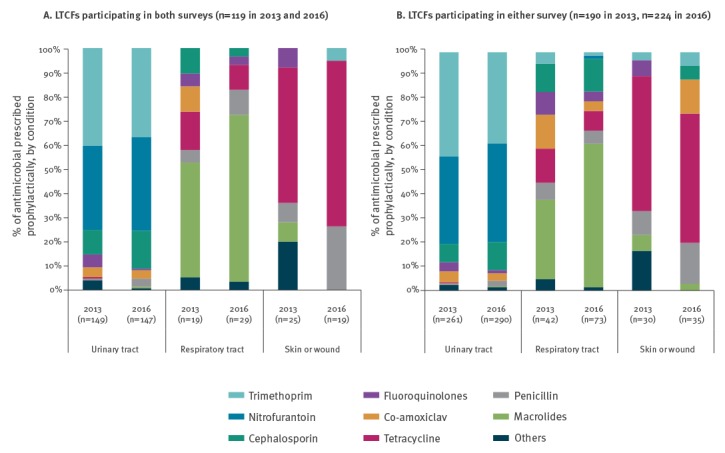
Breakdown of antimicrobial agents’ prophylaxis by top three body sites, healthcare-associated infections in long-term care facilities point prevalence surveys, Ireland, 2013 and 2016

Figure 2 displays a comparison between 2013 and 2016 in the breakdown of prophylactic antimicrobials prescribed according to body site. The analysis of the 119 LTCFs that participated in both surveys, nitrofurantoin (35% and 39%) and trimethoprim (40% and 37%) were most often prescribed as prophylaxis for urinary tract; macrolides (47% and 69%), cephalosporin (11% and 3%) and tetracycline (16% and 11%) for respiratory tract and tetracycline (56% and 68%) and penicillin (8% and 26%) for skin or wounds in respective years (Figure 2A). The prophylactic prescribing of macrolides for respiratory tract and tetracycline for skin or wounds was higher in 2016 compared with 2013.

The analysis of all the LTCFs for the 2 years shows similar results. Nitrofurantoin (37% and 41%) and trimethoprim (44% and 38%) were the most frequently prescribed prophylaxis for urinary tract, macrolides (33% and 60%), cephalosporins (12% and 14%) and tetracycline (14% and 8%) were most often prescribed for respiratory tract prophylaxis in 2013 and 2016 respectively, tetracycline (57% and 54%) and penicillin (10% and 17%) were the main prophylactic agents prescribed for skin or wounds. Tetracycline was mainly prescribed for skin or wound in IDFs (47% and 58%). The proportion of fluoroquinolone prophylaxis in 2013 and 2016 for urinary tract was 4% and 1%, for respiratory tract 10% and 4% and for skin or wounds 7% and 0% (Figure 2B).

### Risk factors of prophylactic prescribing

The median age of residents on prophylactic antimicrobials was 82 years, with an interquartile range of 71–88 years (not shown in table). The number of residents over 85 years on prophylaxis was 30% higher in 2013 (39%, 133/341) compared with 2016 (44%, 174/395). Table 2 displays a higher proportion of females (44%) on antimicrobial prophylaxis than males (35%). Residents who had been in the LTCF for more than 1 year were more likely to be prescribed prophylaxis (46% vs 29%).

**Table 2:** Multiple logistic regression analysis of prophylactic vs therapeutic antimicrobial prescribing in long-term care facilities, Ireland, Healthcare-Associated Infections in Long-Term Care Facilities (HALT) 2013 (n = 190 facilities) and 2016 (n = 224 facilities)

Characteristics		Total	Prophylactic		Multivariate analysis	
			n	%	OR (95% CI)	p value
**Age**	< 85 years	1,123	444	39.5	ref	
	> 85 years	735	307	41.8	1.04 (0.8–1.3)	0.37
**Sex**	Male	696	243	34.9	ref	
	Female	1,162	508	43.7	1.5 (1.2–1.8)	< 0.001**^a^**
**LOS**	< 1 year	604	175	28.9	ref	
	> 1 year	1,254	576	45.9	1.4 (1.1–1.8)	0.004 **^a^**
**Hospital admission ^b^**	No	1,445	649	44.9	ref	
	Yes	413	102	24.7	0.5 (0.4–0.7)	< 0.001 **^a^**
**Urinary catheter**	No	1,603	636	39.7	ref	
	Yes	255	115	45.1	1.8 (1.4–2.5)	< 0.001 **^a^**
**Vascular catheter**	No	1,802	747	41.5	ref	
	Yes	56	4	7.1	0.2 (0.1–0.4)	< 0.001 **^a^**
**Incontinence**	No	648	220	33.9	ref	
	Yes	1,210	531	43.9	1.3 (1.1–1.7)	0.005 **^a^**
**Pressure sore**	No	1,753	722	41.2	ref	
	Yes	105	29	27.6	0.6 (0.4–1.0)	0.03 **^a^**
**Facility type**	GNH	1,034	417	40.3	ref	
	IDF	207	108	52.2	1.5 (1.1–2.2)	0.007 **^a^**
	Mixed	410	162	39.5	1.0 (0.8–1.3)	0.92
	Other	207	64	30.9	1.0 (0.7–1.4)	0.87
**Participation in HALT surveys**	2013 only	382	140	36.6	ref	
	2016 only	461	210	45.5	1.6 (1.2–2.1)	0.002 **^a^**
	Both (2013 and 2016)	1,015	401	39.5	1.2 (1.0–1.6)	0.09

CI: confidence interval; GNH: general nursing home; HALT: healthcare-associated infections in long-term care facilities; IDF: intellectual disability facility; LOS: length of stay in long-term care facility; OR: odds ratio; ref: reference value.

^a ^Indicates significant p value of < 0.05.

^b ^Indicates hospital admission in last 3 months of survey.

The results of multiple logistic regression analysis demonstrated an increased odds of prophylaxis for IDF residents, for those resident more than 1 year in the LTCF, for females, with the presence of a urethral catheter and with incontinence (Table 2). Residents who had a pressure sore or a recent hospital admission were less likely to be on antimicrobial prophylaxis, although pressure sore was not significant when only comparing LTCFs which participated in both HALT surveys.

Prophylactic prescribing was 1.6 times higher in LTCFs that only participated in HALT 2016, compared with those only participating in 2013. LTCFs participating in both surveys showed no difference in prophylaxis prescribing between the surveys (Table 2).

### Unusual prophylactic prescribing in long-term care facilities

There was no documented end date for 80% of prophylactic prescriptions. A high prevalence of antimicrobial prophylaxis was observed in IDFs. In the 2013 PPS, there was one IDF with five male residents (9%) who were prescribed oral vancomycin as skin or wound prophylaxis. In both PPS, there were residents prescribed two different prophylactic antimicrobials (n = 4 in 2013; n = 6 in 2016). Indeed, there was one resident in 2013 simultaneously prescribed three antimicrobials (co-amoxiclav, nitrofurantoin and trimethoprim) as urinary tract prophylaxis. Some residents were simultaneously receiving two different antimicrobial agents, one prophylactic and the other as a therapeutic. Two residents in 2013 received two different types of antimicrobial prophylaxis for the same body site and three residents in 2016 received two types of antimicrobial prophylaxis for different body sites (Table 3).

**Table 3:** Special cases in the studies (prophylactic and therapeutic antimicrobial prescribing)

HALT-2013			HALT- 2016		
Resident	Antimicrobial	Prescribed for	Resident	Antimicrobial	Prescribed for
R13_1	Doxycycline (P)	S/W	R16_1	Azithromycin(P)	RT
	Rifampicin (P)	S/W		Nitrofurantoin(P)	UT
R13_2	Nitrofurantoin(P)	UT	R16_2	Doxycycline(P)	S/W
	Cephalexin(P)	UT		Nitrofurantoin(P)	UT
R13_3	Mycostatin (P)	ENM	R16_3	Nitrofurantoin(P)	UT
	Co-amoxiclav (T)	RT		Doxycycline(P)	S/W
R13_4	Erythromycin(P)	other	R16_4	Cefalexin(P)	UT
	Co-amoxiclav (T)	RT		Trimethoprim(P)	UT
R13_5	Trimethoprim(P)	UT	R16_5	Trimethoprim(P)	UT
	Ciprofloxacin(T)	UT		Co-amoxiclav (T)	RT
R13_6	Mycostatin(P)	ENM	R16_6	Cefaclor(P)	S/W
	Ciprofloxacin(T)	UT		Trimethoprim(T)	RT
R13_7	Trimethoprim(P)	UT	R16_7	Trimethoprim(P)	UT
	Nitrofurantoin(P)	UT		Metronidazole (T)	Other
R13_8	Nitrofurantoin(P)	UT	R16_8	Cefixime(P)	RT
	Trimethoprim(P)	UT		Azithromycin(P)	RT
R13_9	Nitrofurantoin(P)	UT	R16_9	Trimethoprim(P)	UT
	Co-amoxiclav (P)	UT		Co-amoxiclav (T)	RT
	Trimethoprim(P)	UT		Chloromycetin(T)	Eye
R13_10	Trimethoprim(P)	UT	R16_10	Doxycycline(P)	RT
	Cefpodoxime(T)	RT		Cefixime(P)	RT
R13_11	Trimethoprim(P)	UT	R16_11	Cefixime(P)	RT
	Ciprofloxacin(T)	UT		Doxycycline(P)	RT
R13_12	Trimethoprim(P)	UT	R16_12	Nitrofurantoin(P)	UT
	Co-amoxiclav(T)	RT		Co-amoxiclav (T)	RT
R13_13	Azithromycin(P)	RT	NA		
	Trimethoprim(T)	UT			

ENM: ear, nose and mouth; HALT: healthcare-associated infections in long-term care facilities; P: resident is on prophylaxis for particular antimicrobials; RT: respiratory tract; S/W: skin or wounds; T: resident is on therapeutic treatment for particular antimicrobials; UT: urinary tract.

Every serial number (SN) represents a resident as R13_1, R13_2 and so on, R13 refers to residents in the 2013 survey and R16 is a resident in 2016.

## Discussion

These findings showing a high prevalence of prophylactic antimicrobial use in LTCFs are consistent with previous studies conducted in the EU/EEA region [2,3] and in Ireland [5,19-21]. Overall, more than 40% of antimicrobial prescriptions in Irish LTCFs were for prophylaxis. After correcting for demographic factors and the type of facility, prophylactic use of antimicrobials was higher for LTCFs only participating in the 2016 survey, compared with those only participating in 2013. It is unclear why there was more prophylactic prescribing in LTCFs that performed the 2016 survey only. It might be expected that prior participation in the 2013 survey might have increased awareness of high prophylactic prescribing, which in turn may have had an impact on subsequent prophylactic prescribing. Only 119 LTCFs participated in both surveys (2013 and 2016). However, the LTFCs participating in both surveys show no difference in prophylaxis use. More than 90 LTCFs participated for the first time in the HALT survey in 2016 and awareness of prophylaxis prescribing in the new participating LTCFs may have been lower before participating in HALT. Anecdotal feedback received from 2013 HALT participants who attended HALT protocol training sessions in 2016 indicated that prior participation had indeed raised local awareness about antimicrobial stewardship in LTCF nursing staff, although the impact on prescribing doctors was felt to be less evident. In both 2013 and 2016 HALT surveys, LTCF nursing staff comprised the vast majority of HALT data collectors, with very few doctors reported to have collected data (data not shown).

Although there is scientific evidence for antimicrobial prophylaxis in selected indications (e.g. perioperative prophylaxis), much of the prophylactic prescribing in LTCFs does not appear to follow guidelines or be evidence-based [27]. Our other study showed that nearly half of the antimicrobial prescribed in LTCF were broad-spectrum/secondline [28]. In this study, the majority of prophylaxis was prescribed for the urinary tract (75%) and no proportional change was observed between 2013 and 2016. Antimicrobial prophylaxis may be considered for recurrent symptomatic urinary tract infection (UTI) (defined as three or more uncomplicated UTIs in the previous year) if the condition is chronic and impacts on the well-being of a patient [7]. However, the scale of urinary tract prophylaxis in Irish LTCFs suggests a more liberal use of prophylaxis. Positive urine dipsticks and asymptomatic bacteriuria are especially common in older adults and may be erroneously interpreted as representing UTI in residents without convincing localising symptoms [29]. In particular, residents with a urinary catheter were 1.8 times more likely to receive antimicrobial prophylaxis, even though the presence of a urinary catheter is generally deemed a contraindication to prophylaxis [30]. Similarly, a multilevel analysis of the additional collected data from HALT 2016 by the author showed urinary catheter as a key predictor of antimicrobial use in LTCFs [31].

Nitrofurantoin and trimethoprim were the main agents prescribed for urinary tract prophylaxis. Long-term use of nitrofurantoin is not advised, due to its hepatotoxicity [10] and pulmonary, nerve and liver adverse reactions reported as a result of long-term nitrofurantoin prophylaxis in elderly patients [32].

The proportion of antimicrobials prescribed for respiratory tract prophylaxis was higher in 2016 than in 2013 (26% vs 16%). Even though the number of residents aged over 85 years was higher in 2013 compared with 2016, and chronic bronchitis and chronic obstructive pulmonary disease (COPD) are more prevalent in older age groups [33], these conditions do not necessitate prophylactic antimicrobials [34]. Macrolides were the most common prescribed prophylaxis for respiratory tract, mainly azithromycin and erythromycin. This higher prescribing of prophylactic antimicrobials for respiratory tract may be related to increased awareness of evidence showing efficacy of macrolide prophylaxis in achieving a reduction in frequency of exacerbations of chronic obstructive pulmonary disease (COPD) [35]. Azithromycin may be indicated for prolonged exacerbations of COPD but prophylaxis should be initiated only upon the direction of a respiratory physician [7].

Fluoroquinolones are not recommended as first-line agents for treatment of either upper or lower respiratory tract infections or UTIs and should only be used as reserved antimicrobials [7]. This study found a limited number of fluoroquinolones prescribed as prophylaxis (2–5%) and cephalosporins accounted for 10% of prophylaxis. Although a relatively small proportions of all antimicrobials used for prophylaxis, the use of these agents is of more concern because of well-documented *C. difficile* infection, with many LTCF residents having additional risk factors for *C. difficile* infection including advanced age and being immunocompromised [36]. Furthermore, use of fluoroquinolone was more often associated with adverse drug events than any other antimicrobial [37].

The other common indication for prophylactic antimicrobials was skin or wound prophylaxis. In LTCFs, wound infections are generally related to ageing, such as dry, pruritic skin, pressure ulcers, scabies, tinea versicolour and prophylaxis is not indicated for these conditions [38]. However, of the antimicrobials prescribed for skin or wound prophylaxis, 71% were tetracycline, mainly doxycycline. The prophylactic use of doxycycline might be in relation to acne vulgaris for which guidelines outline the long-term use of doxycycline. Prophylactic doxycycline for skin or wound was observed mainly in IDFs, in which residents are generally younger and residents may have chronic skin wounds secondary to self-injurious behaviour [39]. A concerning finding of the 2013 PPS was that five male residents in one IDF were prescribed oral vancomycin for skin or wound prophylaxis.

In Ireland, 70% of nursing home residents are considered to be highly dependent and require long-term care [40]. According to previous studies, residents living in nursing homes for at least 6 months are 40­–70% more likely to receive at least one antimicrobial [8]. The results of this study showed a doubling of antimicrobial prophylaxis in those residing in LTCFs for more than 1 year. This may be explained on the basis that the longer a resident remains in the same LTCF, the more likely the resident is to be potentially erroneously labelled as having recurrent UTIs and for prophylaxis to be considered a management option. A recent hospital admission (within 3 months of the survey) was highly associated with a reduction in antimicrobial prophylaxis, which may be due to a revision or termination of prescriptions during a hospital stay. The fact that some residents in this study were simultaneously prescribed more than one prophylactic antimicrobial highlights the importance of regular medicine reconciliation exercises.

### Limitations

The participation of Irish LTCFs in the HALT surveys was voluntary. LTCFs with an interest in antimicrobial stewardship and HAI prevention may have been more likely to participate. The analysis was performed using data collected as part of a PPS using a standardised data collection form. While more beneficial for data collection, it also limits the opportunity to identify alternative or different interpretations of indications for antimicrobial use or application of guidelines or indications for prophylaxis. This applies to any prophylaxis related to urinary tract, respiratory tract or skin or wounds. Additionally, a PPS does not lend itself to determining duration of antimicrobial courses, as start and end dates cannot be determined.

The risk-factor analysis was limited to residents on antimicrobials where the odds expressed in the paper are the odds of a patient being on a prophylactic antimicrobial compared with being on a therapeutic antimicrobial. This actually results in few significant results which is a limitation resulting from how data were collected in the study. The significance of the result has been reported using the exact p value, while the repeated measurement for adjusting multiple testing should have been considered in the study.

### Conclusion

A higher proportion of prophylactic antimicrobial prescribing was observed in relation to the respiratory tract in 2016 compared with 2013 in Irish LTCFs. Overall, no differences in prophylactic antimicrobial prescribing were observed among the LTCFs participating in both HALT surveys. The use of prophylaxis for the urinary tract remained high and unchanged. The high prevalence of antimicrobial use in Irish LTCFs, particularly antimicrobial prophylaxis, indicates an urgent need to develop and implement education and stewardship programmes specifically targeted towards residential care settings. While participation in repeated HALT surveys is a valuable surveillance method, it must be supplemented by local quality improvement initiatives, based on each LTCF’s survey results.

## References

[1] World Health Organization (WHO). Antimicrobial resistance: global report on surveillance 2014. Geneva: WHO; Apr 2014. Available from: https://www.who.int/drugresistance/documents/surveillancereport/en/

[2] European Centre for Disease Prevention and Control (ECDC). Point prevalence survey of health care-associated infections and antimicrobial use in European long-term care facilities. April–May 2013. Stockholm: ECDC; 2014. Available from: https://ecdc.europa.eu/en/publications-data/point-prevalence-survey-healthcare-associated-infections-and-antimicrobial-use-2

[3] European Centre for Disease Prevention and Control (ECDC). Point prevalence survey of healthcare-associated infections and antimicrobial use in European long-term care facilities. May–September 2010. Stockholm: ECDC; 2010. Available from: https://ecdc.europa.eu/en/publications-data/point-prevalence-survey-healthcare-associated-infections-and-antimicrobial-use-1

[4] Ricchizzi E Latour K Kärki T Buttazzi R Jans B Moro ML Antimicrobial use in European long-term care facilities: results from the third point prevalence survey of healthcare-associated infections and antimicrobial use, 2016 to 2017. Euro Surveill. 2018;23(46):1800394. 10.2807/1560-7917.ES.2018.23.46.1800394 30458913 PMC6247460

[5] Health Protection Surveillance Centre (HPSC). Point prevalence survey of healthcare‐associated infections & antimicrobial use in long‐term care facilities (HALT): May 2013. National report 2013. Dublin: HPSC; Mar 2014. Available from: https://www.hpsc.ie/a-z/microbiologyantimicrobialresistance/infectioncontrolandhai/surveillance/hcaiinlongtermcarefacilities/haltreports/2013report/national2013haltreport/File,14540,en.pdf

[6] National Institute for Health and Care Excellence (NICE). Antibiotic use. London: NICE; 2016. Available from: https://www.nice.org.uk/guidance/conditions-and-diseases/infections/antibiotic-use

[7] Health Service Executive (HSE). Guidelines for Antimicrobial Prescribing in Primary Care in Ireland. Dublin: HSE; 2012. Available from: http://www.antibioticprescribing.ie/

[8] Crnich CJ Jump R Trautner B Sloane PD Mody L Optimizing antibiotic stewardship in nursing homes: a narrative review and recommendations for improvement. Drugs Aging. 2015;32(9):699-716. 10.1007/s40266-015-0292-7 26316294 PMC4579247

[9] Brown KA Khanafer N Daneman N Fisman DN Meta-analysis of antibiotics and the risk of community-associated Clostridium difficile infection. Antimicrob Agents Chemother. 2013;57(5):2326-32. 10.1128/AAC.02176-12 23478961 PMC3632900

[10] Cetti RJ Venn S Woodhouse CRJ The risks of long-term nitrofurantoin prophylaxis in patients with recurrent urinary tract infection: a recent medico-legal case. BJU Int. 2009;103(5):567-9. 10.1111/j.1464-410X.2008.08008.x 18782308

[11] Vellinga A Cormican M Hanahoe B Murphy AW Predictive value of antimicrobial susceptibility from previous urinary tract infection in the treatment of re-infection. Br J Gen Pract. 2010;60(576):511-3. 10.3399/bjgp10X514765 20594440 PMC2894379

[12] Ellis C. Long-term antibiotic use may lead to increased risk of cancer. Cranbury: Contagion; 11 Apr 2017. Available from: http://www.contagionlive.com/news/long-term-antibiotic-use-may-lead-to-increased-risk-of-cancer#sthash.nmo5WcqW.dpuf

[13] Enzler MJ Berbari E Osmon DR Antimicrobial prophylaxis in adults. Mayo Clin Proc. 2011;86(7):686-701. 10.4065/mcp.2011.0012 21719623 PMC3127564

[14] Eurostat. EU in the world. 2016 edition. Luxembourg: Publications Office of the European Union; 2016. Available from: https://ec.europa.eu/eurostat/web/products-statistical-books/-/KS-EX-16-001

[15] Rechel B, Grundy YDE, McKee M. How can health systems respond to population ageing? Copenhagen: World Health Organization on behalf of the European Observatory on Health Systems and Policies; 2009. Available from: http://www.euro.who.int/__data/assets/pdf_file/0004/64966/E92560.pdf

[16] Suetens C Healthcare-associated infections in European long-term care facilities: how big is the challenge? Euro Surveill. 2012;17(35):20259. 22958606

[17] Rowe T, Iyer G. Challenges to diagnosis and management of infections in older adults. In: Lindquist LA, editor. New Directions in Geriatric Medicine: Concepts, Trends, and Evidence-Based Practice. Cham: Springer International Publishing; 2016. p. 31-47.

[18] Norman DC Clinical features of infection in older adults. Clin Geriatr Med. 2016;32(3):433-41. 10.1016/j.cger.2016.02.005 27394015

[19] Health Service Executive (HSE). European point prevalence survey on healthcare-associated infections and antibiotic use in long‐term care facilities. National report – Republic of Ireland. Dublin: HSE; Nov 2010. Available from: https://www.hpsc.ie/a-z/microbiologyantimicrobialresistance/infectioncontrolandhai/surveillance/hcaiinlongtermcarefacilities/haltreports/2010report/File,4723,en.pdf

[20] Health Protection Surveillance Centre (HPSC). Point prevalence survey of healthcare-associated infections & antimicrobial use in long-term care facilities: May 2016, Ireland National report. Dublin: HPSC; Mar 2017. Available from: http://www.hpsc.ie/a-z/microbiologyantimicrobialresistance/infectioncontrolandhai/surveillance/hcaiinlongtermcarefacilities/haltreports/2016report/File,16218,en.pdf

[21] Health Service Executive (HSE). Second national prevalence survey on healthcare-associated infections and antibiotic use in Irish long-term care facilities-National report. Dublin: HSE; Aug 2011. Available from: http://www.hpsc.ie/a-z/microbiologyantimicrobialresistance/infectioncontrolandhai/surveillance/hcaiinlongtermcarefacilities/haltreports/2011report/File,12869,en.pdf

[22] European Centre for Disease Prevention and Control (ECDC). Protocol for point prevalence surveys of healthcare-associated infections and antimicrobial use in European long-term care facilities. Version v.2014. Stockholm: ECDC; 2014. Available from: https://ecdc.europa.eu/sites/portal/files/media/en/publications/Publications/healthcare-associated-infections-point-prevalence-survey-long-term-care-facilities.pdf

[23] McGeer A Campbell B Emori TG Hierholzer WJ Jackson MM Nicolle LE Definitions of infection for surveillance in long-term care facilities. Am J Infect Control. 1991;19(1):1-7. 10.1016/0196-6553(91)90154-5 1902352

[24] Stone ND Ashraf MS Calder J Crnich CJ Crossley K Drinka PJ Surveillance definitions of infections in long-term care facilities: revisiting the McGeer criteria. Infect Control Hosp Epidemiol. 2012;33(10):965-77. 10.1086/667743 22961014 PMC3538836

[25] R Core Team. A language and environment for statistical computing. Vienna, Austria: R Foundation for Statistical Computing; 2016.

[26] Health Protection Surveillance Centre (HPSC). Healthcare-associated infections & antimicrobial use in long-term care facilities (HALT) May 2016 Survey Protocol – Version 1.0. Dublin: HPSC; 2016. Available from: https://www.hpsc.ie/a-z/microbiologyantimicrobialresistance/infectioncontrolandhai/surveillance/hcaiinlongtermcarefacilities/documentationandsoftwareforundertakinghalt/File,15614,en.pdf

[27] Wolf JS Jr Bennett CJ Dmochowski RR Hollenbeck BK Pearle MS Schaeffer A Best practice policy statement on urologic surgery antimicrobial prophylaxis. J Urol. 2008;179(4):1379-90. 10.1016/j.juro.2008.01.068 18280509

[28] Tandan M Burns K Murphy H Hennessy S Cormican M Vellinga A Improving Antimicrobial Prescribing: A Multinomial Model Identifying Factors Associated With First- and Second-Line Prescribing. J Am Med Dir Assoc. 2018;S1525-8610(18)30608-X. 10.1016/j.jamda.2018.10.028 30554988

[29] Rowe TA Juthani-Mehta M Urinary tract infection in older adults. Aging Health. 2013;9(5):519-28. 10.2217/ahe.13.38 24391677 PMC3878051

[30] Health Protection Surveillance Centre (HPSC). Guidelines for the prevention of catheter-associated urinary tract infection. Published on behalf of a Strategy for the Control of Antimicrobial Resistance in Ireland (SARI). Dublin: HPSC; 2011. Available from: https://www.hpsc.ie/a-z/microbiologyantimicrobialresistance/infectioncontrolandhai/guidelines/File,12913,en.pdf

[31] Tandan M Burns K Murphy H Hennessy S Cormican M Vellinga A Antimicrobial prescribing and infections in long-term care facilities (LTCF): a multilevel analysis of the HALT 2016 study, Ireland, 2017. Euro Surveill. 2018;23(46):1800278. 10.2807/1560-7917.ES.2018.23.46.1800278 30458910 PMC6247462

[32] Rego LL Glazer CS Zimmern PE Risks of long-term use of nitrofurantoin for urinary tract prophylaxis in the older patient. Urol Sci. 2016;27(4):193-8. 10.1016/j.urols.2016.07.004

[33] Kim V Criner GJ Chronic bronchitis and chronic obstructive pulmonary disease. Am J Respir Crit Care Med. 2013;187(3):228-37. 10.1164/rccm.201210-1843CI 23204254 PMC4951627

[34] Woodhead M Blasi F Ewig S Garau J Huchon G Ieven M Guidelines for the management of adult lower respiratory tract infections-full versio. Clin Microbiol Infect. 2011;17(s6):E1-59. 10.1111/j.1469-0691.2011.03672.x 21951385 PMC7128977

[35] Aaron SD. Management and prevention of exacerbations of COPD. BMJ. 2014;349(sep22 3):g5237. 25245156 10.1136/bmj.g5237

[36] Dancer SJ The problem with cephalosporins. J Antimicrob Chemother. 2001;48(4):463-78. 10.1093/jac/48.4.463 11581224

[37] Tandan M Cormican M Vellinga A Adverse events of fluoroquinolones vs. other antimicrobials prescribed in primary care: A systematic review and meta-analysis of randomized controlled trials. Int J Antimicrob Agents. 2018;52(5):529-40. 10.1016/j.ijantimicag.2018.04.014 29702230

[38] Montoya A Mody L Common infections in nursing homes: a review of current issues and challenges. Aging Health. 2011;7(6):889-99. 10.2217/ahe.11.80 23264804 PMC3526889

[39] Roche FM Donlon S Burns K Point prevalence survey of healthcare-associated infections and use of antimicrobials in Irish intellectual disability long-term care facilities: 2013. J Hosp Infect. 2016;93(4):410-7. 10.1016/j.jhin.2016.03.006 27112050

[40] Drennan J, Lafferty A, Treacy MP, Fealy G, Phelan A, Lyons I, et al. Older people in residential care settings. Results of a national survey of staff-resident interactions and conflicts. Dublin: NCPOP, University College Dublin; 2012. Available from: http://www.ncpop.ie/userfiles/file/Older%20People%20in%20Residential%20Care%20Settings_Final%20Proof_28Nov2012.pdf

